# A digital repository with an extensible data model for biobanking and genomic analysis management

**DOI:** 10.1186/1471-2164-15-S3-S3

**Published:** 2014-05-06

**Authors:** Massimiliano Izzo, Francesco Mortola, Gabriele Arnulfo, Marco M Fato, Luigi Varesio

**Affiliations:** 1 Department of Computer Science Bioengineering Robotics and Systems Engineering, University of Genoa, Viale Causa 13, 16145 Genoa, Italy; 2 Laboratory of Molecular Biology, Giannina Gaslini Institute, Largo Gaslini 5, 16147 Genoa, Italy; 3 Neuroscience Center, P.O. Box 56, FI-00014 University of Helsinki, Finland

## Abstract

**Motivation:**

Molecular biology laboratories require extensive metadata to improve data collection and analysis. The heterogeneity of the collected metadata grows as research is evolving in to international multi-disciplinary collaborations and increasing data sharing among institutions. Single standardization is not feasible and it becomes crucial to develop digital repositories with flexible and extensible data models, as in the case of modern integrated biobanks management.

**Results:**

We developed a novel data model in JSON format to describe heterogeneous data in a generic biomedical science scenario. The model is built on two hierarchical entities: processes and events, roughly corresponding to research studies and analysis steps within a single study. A number of sequential events can be grouped in a process building up a hierarchical structure to track patient and sample history. Each event can produce new data. Data is described by a set of user-defined metadata, and may have one or more associated files. We integrated the model in a web based digital repository with a data grid storage to manage large data sets located in geographically distinct areas. We built a graphical interface that allows authorized users to define new data types dynamically, according to their requirements. Operators compose queries on metadata fields using a flexible search interface and run them on the database and on the grid. We applied the digital repository to the integrated management of samples, patients and medical history in the BIT-Gaslini biobank. The platform currently manages 1800 samples of over 900 patients. Microarray data from 150 analyses are stored on the grid storage and replicated on two physical resources for preservation. The system is equipped with data integration capabilities with other biobanks for worldwide information sharing.

**Conclusions:**

Our data model enables users to continuously define flexible, ad hoc, and loosely structured metadata, for information sharing in specific research projects and purposes. This approach can improve sensitively interdisciplinary research collaboration and allows to track patients' clinical records, sample management information, and genomic data. The web interface allows the operators to easily manage, query, and annotate the files, without dealing with the technicalities of the data grid.

## Background

Data management and integration has become a major issue in contemporary biomedical research. Modern genomic profiling platforms, such as high-throughput gene sequencing platforms, now produce outputs of several hundreds of gigabases. The gathered genomic information must be integrated with all available data about patient clinical history and lifestyle. This unified overview will be of paramount importance as healthcare paradigms move towards personalized medicine. Extensive metadata are required to improve the collection and analysis of this information. For these same reasons, life science research is evolving into international multi-disciplinary collaborations based upon increasing data sharing among labs and institutions. Individual labs implement different protocols and perform their analyses using different instruments. Therefore, metadata are inconsistent, poorly defined, ambiguous and do not use a common vocabulary or terminology. Research collaborations are evolving from local to global scales, the heterogeneity of the collected metadata grows and no single standardization is possible. For this reason, a flexible and extensible metadata model for data integration and sharing now takes fundamental significance. Biomedical researchers proposed different models as the core functionality of data management systems to deal with this issue. Some of these systems have proved to be useful in large-scale projects, and the metadata model is often related to the standard format used [1]. MIBBI [2] and MIAME [3] have been developed in compliance to community standards in different biology areas. SysMO-SEEK [4] provides the most elaborated approach for automated data collection: harvesters are automatically looking for new data and feeding it to the system. Semi-automated approaches are the dropboxes of openBIS [5] and batch import facilities in most other systems, like Gaggle-BRM [6], MIMAS [7], XperimentR [8], ISA tools [9], BASE [10], LabKey [11]. In the context of next generation sequencing integration, openBIS enables users to collect, integrate, share, publish data and to connect to data processing pipelines. This framework can be extended and has been customized for different data types acquired by a range of technologies. In DIPSBC [12] standard data types are described by writing XML indexed files. The XML-based Clinical and Experimental Data Exchange (XCEDE) schema provides an extensive metadata hierarchy for storing, describing and documenting data generated by scientific studies [13]. XCEDE hierarchical structure models scientific experiments using entities such as projects, subjects, studies, visits and acquisitions and allows to track patients clinical history. XCEDE is more suitable to exchange documents and protocols about research projects than to share the data themselves. The Functional Genomics Experiment data model (FuGE) [14] aims to develop a standard for data sharing. It is used to describe complete experimental workflows and it relies heavily on inheritance and ontologies. Solutions for integrating resources and solving databases interoperability often consist in combining web services [15-18] with data warehousing systems and federated databases, using sophisticated tools like BioMart, MOLGENIS and Taverna Workbench [19]. BioMOBY is an open source ontology-based integration system for accessing distributed and heterogeneous data sources via web services [20]. The BioRegistry repository is a relational database that provides classification of the data sources according to shared metadata [21-24]. All these systems allow a single machine to collect all annotations from multiple distributed data sources and display them to the user in a single view. The classification of metadata is used also to manage information about clinical activities [25]. For this reason Busch and Wedemann [26] developed a flexible software framework to fully describe the molecular biology domain. Although a number of current efforts have been devoted to data integration, there is not a optimal general solution yet [15]. One concern with these data model is that they focus either on the clinical or the genomic details and are not suited to describe multi-disciplinary data integration. Modern biobanks face many of the issues mentioned above. In recent years, biobanks have evolved from centres collecting tissues and blood samples, to institutions gathering also a whole spectrum of information including social, clinical, and pathological records together with genomic profiles. Biobanks require robust management systems able to track all the activities, store and annotate all the related data. Thus, a biobank scenario is an ideal test-bed for a metadata model designed to handle heterogeneous data for integration purposes. Numerous efforts have been paid to encourage and propose standards for biobanking integration. The BBMRI consortium defined a minimum data set for sharing biobank samples (MIABIS) [27]. However, not all the biobanks may be able to retrieve all the required information, depending on internal regulations. There exist numerous software solutions developed for integrated biobanking management, such as caTissue [28], SIMBioMS [29] and data warehouses based on the i2b2 platform [30,31]. While some of them provide some level of integration between sample management and clinical or genomic information, they do not supply a flexible metadata model for user customization.

The eXTENsible platform for biomedical Sciences (XTENS) digital repository was originally developed by Corradi et al., at the Department of Informatics Bioengineering Robotics and Systems Engineering (DIBRIS) of the University of Genoa to support integrated research in Neuroscience, with a particular focus on Neuroimaging [32]. Its data management paradigm was designed to handle a various range of situations and environments in Biomedical Research and already incorporated a basic sample management system. We used the XTENS core structure as a backbone to implement and test our data model for biobanking management and functional genomics.

In this paper, we first describe how to incorporate a flexible and extensible data model written in JavaScript Object Notation (JSON) inside the XTENS repository to support heterogeneous data management in a generic biomedical science scenario. Then, we focus on the specific use case of an integrated biobanking management, where different information sources (clinical, histopathological, genomic...) must be queried, integrated, and shown in a structured view.

## Methods

### XTENS Digital Repository

The XTENS repository consists of (1) a web portal, (2) an internal database, (3) and a data grid storage element.

The XTENS portal provides a web interface and allows users to access and manage database requests. We have redesigned the portal to make patient, sample, and data management easier for the laboratory operators. The XTENS portal is a Java Server Pages (JSP) and servlet application deployed on an Apache Tomcat servlet container running on a Linux server. To better enhance user experience and interactivity, various components are designed using Asynchronous JavaScript and XML (AJAX) programming technique. Client and server exchange messages using JSON [33] through JSON-RPC protocol whenever possible. We have built a set of RESTful web services for interaction between XTENS and external application without accessing the web portal to perform *Create-Retrieve-Update-Delete *(CRUD) operations on the database entities.

The repository currently relies on a MySQL 5.5 database. Database access from the web application is managed with MyBatis [34], a persistence framework that automates mapping between SQL databases and Java objects. The MyBatis persistence layer permits us to adopt, if required, a different SQL RDMS (PostgreSQL, Oracle, ...) with moderate effort. The database contains all the information about projects, patients, data and everything related to the repository management (users, groups and accesses). The grid storage element contains all the files associated to registered data instances. The administrator can set up the XTENS system to store metadata both on the internal database and on the grid storage metadata catalogue, or only on one of the two systems. The users and the administrator access the system using an existing LDAP or database account available on the server. Each user is associated to Access Control Lists in order to guarantee security and auditing. The access is via web browser without any client installation and in a secure way through the HTTPS protocol. Authentication and access-control is managed using the Spring Security framework. XTENS addressed possible security and privacy policies regarding the access to proprietary data and sensible clinical data. This is achieved by a thorough customization of user permissions, defined by functions. Authenticated users are allowed to view, insert, modify and retrieve data according with the set of functions enabled for their own group. System administrators are able to define different groups of users associated with different access permission to different pages and functions of the XTENS repository.

### Integrated Rule-Oriented Data System (iRODS) middleware

Researchers from many different science disciplines require handling large and geographically distributed data sets for international collaborations [35]. In computational genomic, the scale of produced data per cost unit has grown at a pace faster than the Moore's law since 2008 [36], making the storage and subsequent analysis of these datasets an increasingly difficult task to tackle. The conjunction of these issues makes data management extremely complicated in such a computational environment. Computational and data grids are specifically designed to deal with all these issues. A Grid is a loose network of computers and storage resources. Computational grids offer a distributed environment to divide up a large computational task among individual machines. Users do not have to worry about the different local hardware specification. Data grids are distributed and heterogeneous storage environments that allow users to store data in different locations with different storage devices without any need for them to be aware of specific low-level data access mechanisms.

iRODS [37] is an open source data grid middleware developed by the Data Intensive Cyber Environments (DICE) research group. An autonomous iRODS system - named iRODS Zone - is constituted by at least an iRODS Server, a metadata catalogue and a Rule Engine Server. The metadata catalogue, named iCAT, is a relational database that describes and locates data objects (i.e. files) within the storage system. Files are actually stored in multiple vaults, i.e. directories located within the physical storage resources. These resources are repositories from which the iCAT server, which is used for mapping the location of logical and physical files, can extract files on request. IRODS currently supports three different storage resources: 'Unix File System', 'HPSS' and 'Amazon S3'. The Rule Engine allows data to be managed with policies expressed as computer actionable rules. The Rule Engine interprets the rules and performs a series of operations using microservices, a set of C language functions with a standard interface. Among others, users can set up rules to automatically handle data preservation, replication, consistency checks of the stored data collections and safe trash management. iRODS provides users with different client interfaces to access the data grid system: (i) *icommands*, a Unix-like command line utilities; (ii) a set of APIs for Java, PHP, and Python, named Jargon, PRODS, and PyRODS respectively; (iii) and a dropbox-like graphical environment named iDROP available as a web or desktop applications. Unfortunately, the graphical tools are minimal and do not allow users to perform the full set of functionalities of iRODS, while the *icommands *are more indicated for iRODS administrators than general users. To simplify the user interaction with the data grid we have embedded in the XTENS web application a set of Java methods based on Jargon to store, retrieve, and tag files with user-defined metadata. In our current implementation, we deal with files whose size is in the order of the tens of MB. Users currently upload these files to the application using an upload dialog box, then the application writes the file on the data grid. In the future we plan to use XTENS also to archive whole genome sequencing data. For that we will develop a background daemon to provide bulk ingestion of huge files (i.e. > 2 GB) using GridFTP protocol; metadata will be registered on the XTENS repository once the file has been stored on the iRODS server, parsing the file header.

## Results

### Data Model implementation

We designed and implemented a flexible and extensible data model, which we have embedded in XTENS. As a first step, we restructured and enhanced the process-event model proposed by Corradi et al. [32] to make it more suitable to handle both clinical and genomic information.

**Figure 1:**
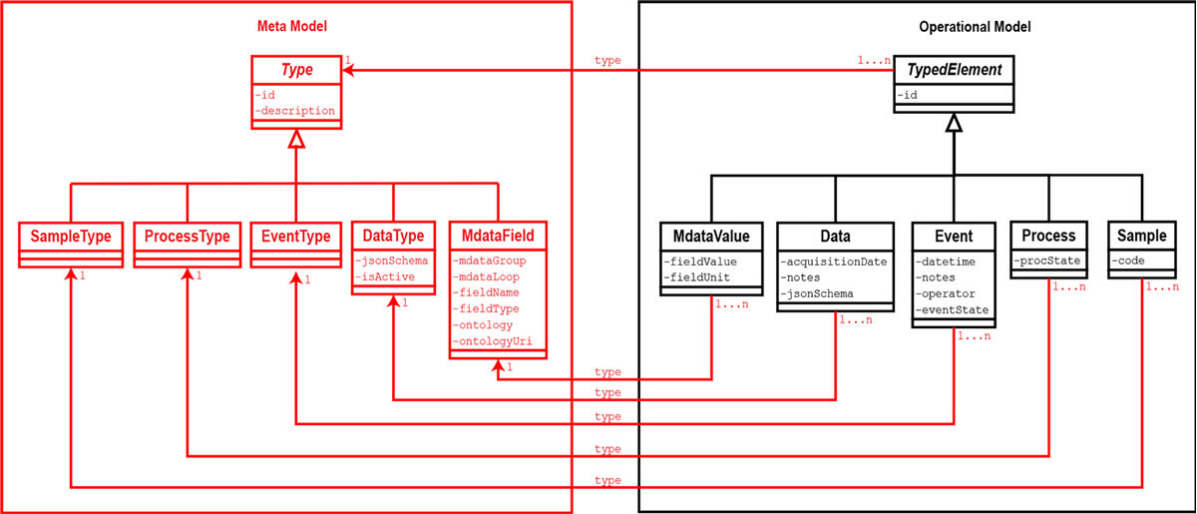
**UML class diagram detailing relations between operational model and meta model**. Details of the class model, showing the operational model (right, in black), which contains all classes involved in the biomedical workflow and the meta model (left, in red) representing the types. All typed classes in the operational Model inherit from TypedElement, while all types in the metaModel inherit from Type. The property *jsonSchema *of DataType contains the metadata schema template as JSON for the specific data type. MdataField represents a metadata attribute definition. Its properties contains info about the metadata group and loop (if there is a loop) the attribute belongs to, its name and type ('STRING', 'INTEGER' or 'FLOAT') and possible ontology references. See the metadata model of Figure 3 for further details. The property *jsonSchema *of Data contains the metadata schema template populated with values (and units) selected by the operator when the data instance was registered in the system. MdataValues represents a single metadata attribute instance, and has properties for value and unit. Event and Process both have a property (*eventState *and *procState*) to check whether they are still active, terminated or paused. The timestamp property *datetime *of Event tracks when a specific event was recorded in the database. The full list of Sample properties is not shown in the picture. Each class in the diagram realizes an XTENS database table.

**Figure 2:**
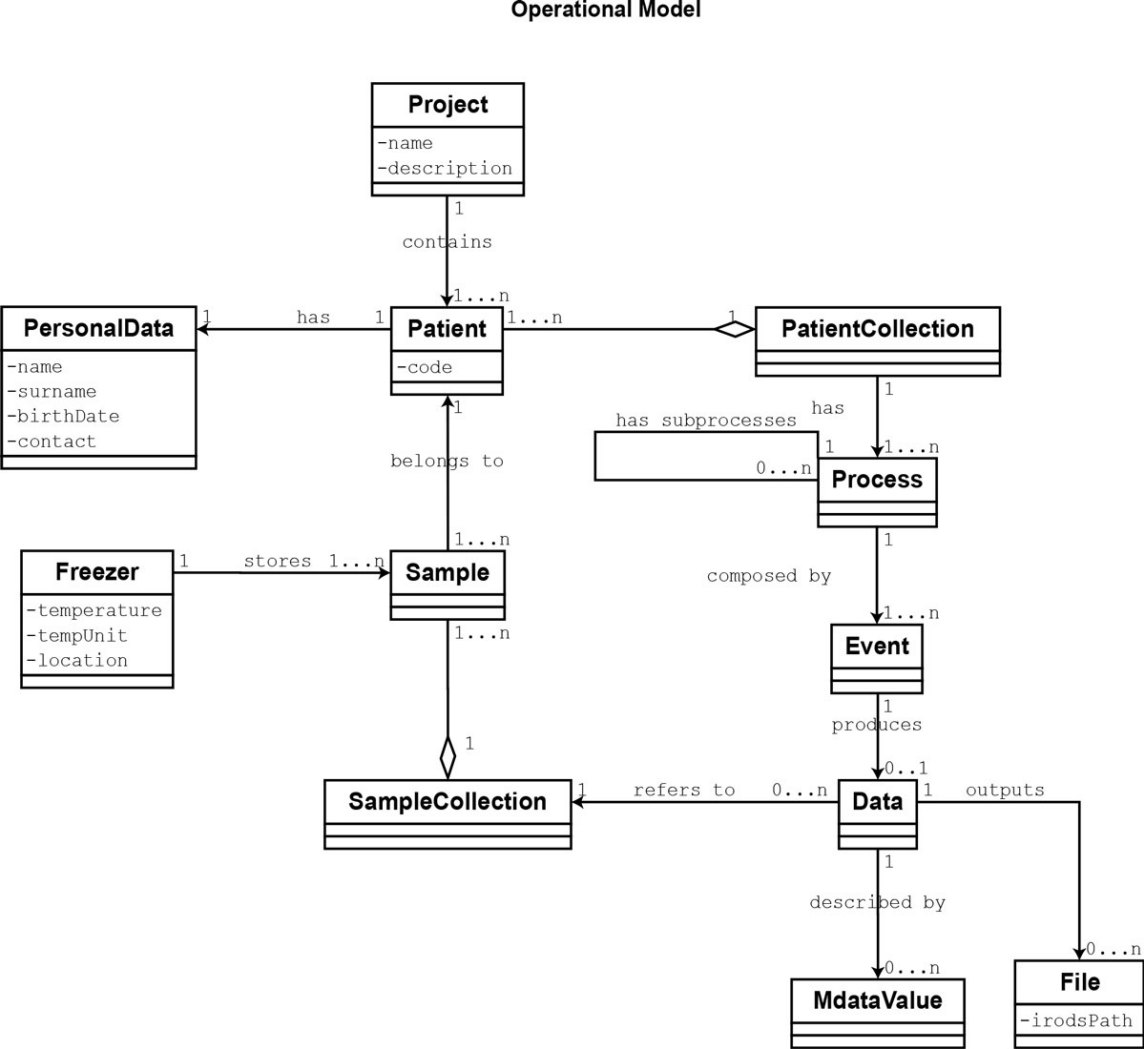
**UML class diagram of the operational model**. The diagram shows the main entities of the biomedical operative model. The Project class represents the top-level component of our model. A PersonalData class contains all the sensitive information about a patient. Only authorized operators can access the identifier that maps Patient objects to PersonalData. The details of the freezer management system are not shown. A process contains sub-processes and/or events, outlining a flexible hierarchical structure. Each Data object maps to a single Event and is described by a set of MdataValues objects. The property *irodsPath *of File contains the logical path of the document in the iRODS file system.

We redesigned the XTENS object model, distinguishing it into two different sub-models (persistent classes are written in Monocode font throughout the text) (see Figures 1,2):

1. The **operational model**, a traditional object-oriented (OO) class model where all the classes that map operational entities of the biomedical domain are defined, together with their associations. A subset of these classes - those shown in Figure 1, namely Sample, Process, Event, Data and MdataValue - are typed classes. All typed classes extend the abstract TypedElement class;

2. The **meta model**, an OO class model that contains all the type definition classes. All the type definition classes implement the abstract Type class;

Therefore, each typed class instance is univocally defined by its type. The concepts of meta and operational models are borrowed, though applied in a different context, from the work of Bush et al. [26] In our operational model, the Project is a macro group where all kind of patients' data and information are collected. Each project contains one or more Patient entities. Once a patient is enrolled in a project, she may enter in a research study, composed by a (maybe flexible) set of analysis steps. The same patient may be involved in more studies, such as a gene expression profiling and/or a clinical trial. The process-event model abstracts the concepts of research studies, experiments and analysis steps using two entities: Process and Event. Our data model is built on these two entities. An event is any 'atomic operation' performed on patients and samples, any processing of data or everything else related to the repository administration and management. A process is a collection of sequential events and/or sub-processes related to an activity, allowing the design of a multi-layered hierarchical structure. We created the entity PatientCollection as an aggregate of Patient, to decouple patients from processes and manage situations where a process (i.e. study) contains analyses that require merging data coming from more than one patient.

**Figure 3:**
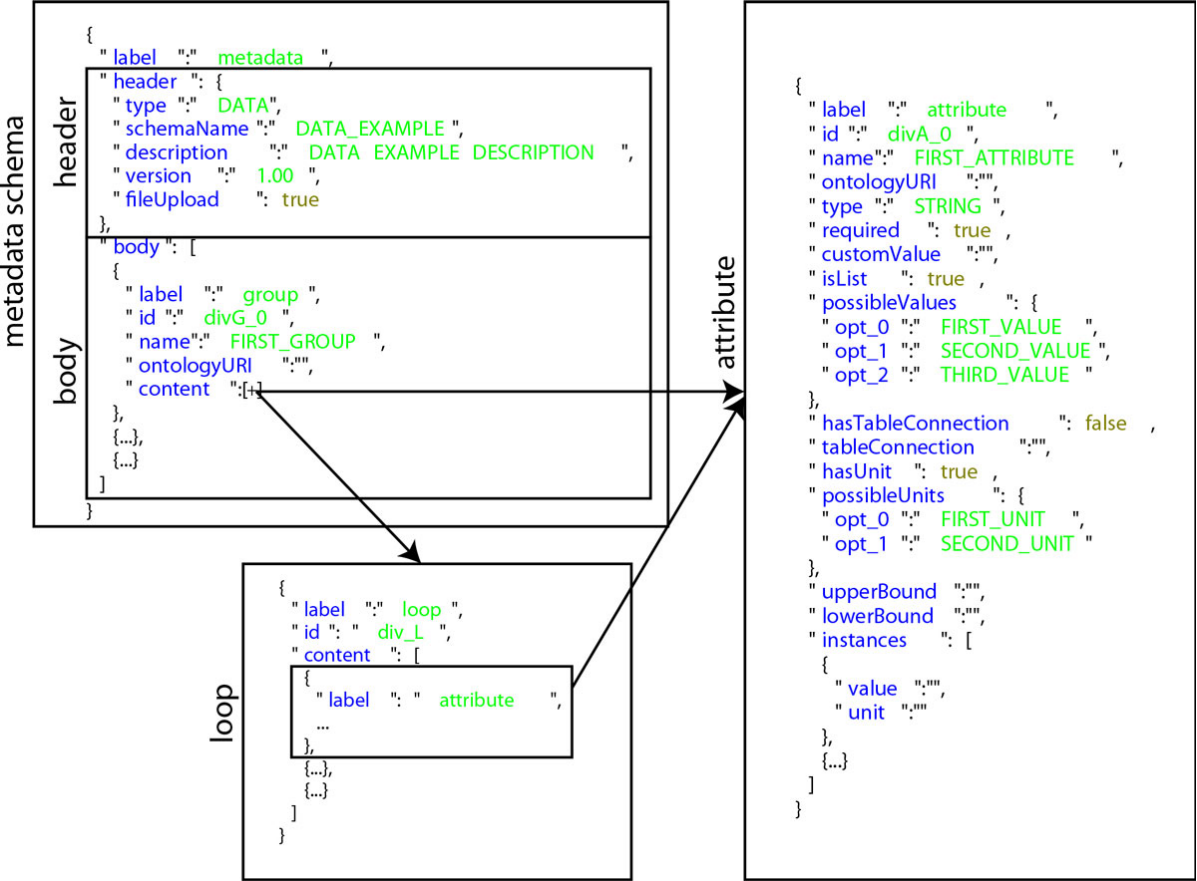
**Metadata model instance**. The metadata JSON schema consists of a header and a body. A metadata body is an array of metadata groups; each group contains attributes (non recursive fields) and/or loops (recursive fields constituted by one or more attributes). Each attribute is a JSON object described by a set of properties to define its type ('STRING', 'INTEGER' or 'FLOAT'), name, if a value and a unit value is required, an optional custom value. A list of possible values and possible units can be stored as lists. If the attribute type is numeric ('INTEGER' or 'FLOAT') users can specify minimum and maximum allowed values using the *lowerBound *and *upperBound *properties. It is possible to name attributes using ontology terms and storing the corresponding URI in the property *ontologyURI*. If the Boolean property *hasTableConnection *is set to true and a *tableConnection *term is defined, the set of possible values is recovered from the XTENS repository internal database. The *instances *property contains all the attribute values and units assigned by the user.

Process and Event objects are fully characterized by the corresponding ProcessType and EventType instances as defined in the meta model. The UML class model of the XTENS core system is shown in Figure 2. Each class shown in the model is persistent and realizes a database entity (i.e. a table). Each time a patient enters a study a new process is activated. A study is composed by a sequence of events, and each of them may produce a Data instance. The structure of a Data instance is defined in the meta model by its DataType, and it is described by a set of user-defined metadata. This is the crucial point of the data model. Metadata are saved as a JSON schema inside a Data entity, where all the metadata properties are written as key-value pairs. Originally, XTENS came with an XML metadata schema. The JSON metadata model is a novel implementation that we have devised to improve schema flexibility and quick parsing. The metadata model details are shown in Figure 3. It consists of two components: a header and a body. The header contains general information about the schema, such as the data type name, a brief description, a boolean term, named *fileUpload*, stating whether or not the data type support file submission, and a serial number for versioning control. The metadata body is a JavaScript array containing one or more metadata group. We introduced the concept of metadata groups to divide metadata on the basis of their type. For instance, users may want to identify the following metadata groups: system metadata, technical metadata on the reproducibility of the experiment, metadata on the operator performing the task (i.e. event), and descriptive metadata retrieved from a file they are storing. Each group can contain fields in the form of attributes, loops or a combination of both. An attribute represents the basic element of the model describing a single, non-recursive metadata field. An attribute is a JavaScript object described by a set of properties (as shown in Figure 3) that can be easily adapted and/or extended. The properties allow determining, among others, the attribute name, type, value and measure unit. Metadata group and attributes can be named after terms selected from an ontology, storing the corresponding *Uniform Resource Identifier *(URI) in a specific property. A different ontology may be used for each data type definition, allowing the coherent integration of multiple terminologies within the same repository.

A loop represents a collection of recursive fields (i.e. attributes). It is useful to store recurrent metadata fields occurring an unforeseeable number of times. Some examples are the field 'metastatic site' for a data type associated to a 'relapse' clinical event, or the 'overexpressed gene' field for a 'DNA microarray' data type. Both of them may occur more than once in a specific data instance, but the number of occurrences varies from time to time. The JSON metadata model also supports nested loops, that can be useful to describe complex genomic data, but we have not employed them so far.

In the XTENS repository authorized users can create and activate on the fly new process, event and data types, without the need to recompile the application. A form-based graphical interface (containing all the fields that map to properties in the metadata JSON schema) allows users/operators to manually define new data types, adding metadata groups, attributes and loops. Users can select an ontology from a list, if they want to name the metadata fields using controlled and semantically associated terms. The selected ontology will be loaded and the application will suggest the terms to the operator using an autocomplete widget. All the attribute parameters and properties specified in the schema (Figure 3) can be set from the form-based interface. A client-side JavaScript procedure scans all its fields when the form is submitted, builds the JSON schema and sends it to the server, which stores the newly created data type as a DataType instance in the XTENS database. The metadata schema is stored in the property *jsonSchema *of DataType. Additionally XTENS stores each attribute definition as a type in the MdataField table. Once the data type is activated for a user group, users belonging to the group can select it to save its data instances. The associated event is first created and inserted in the appropriate process when a user wants to register a new data instance in the repository. Then the metadata schema from the selected data type is retrieved from the database, is parsed, and dynamically converted to a web form using jQuery.dForm [38], a jQuery plugin. When a user submits a data web form, a new Data entity is inserted in the XTENS database. The metadata schema, populated with the values selected by the user, is stored in the property *jsonSchema *of the Data entity (see Figure 2). On submission each metadata attribute is parsed, and its value and measure unit are saved in the MdataValue table of the XTENS database for cataloguing and rapid search. If the *fileUpload *option is set to true in the *jsonSchema *header, one or more files can be uploaded by the user and registered in the in the XTENS data grid system managed by the iRODS middleware. A new file collection is created on iRODS, all the uploaded files are stored within it and the metadata are stored as attribute-value-unit (AVU) triples on the iRODs metadata catalogue (iCAT) and associated to each file in the collection. We also save the JSON schema as a text file in the same iRODS collection where all related data files are saved. This way, in a virtual community scenario involving many institutions, both files and the metadata description could be replicated and shared among all the centres deploying an iRODS server.

A patient can have one or more samples associated to it; we introduce the typed class Sample in the operational model and the associate type SampleType in the meta model (see Figure 2). We performed a further modification to the data management policy enabling data instances association to samples as well as patients. This is crucial in a scenario where researchers want to perform the same functional genomic analysis (e.g. microarray or whole genome sequencing) on DNA or RNA extracted from two different samples (e.g. lymphocytes and tumour tissue) belonging to the same patient, to compare the genomic profile of sane and diseased cells. In such a situation, they must be able to associate each data instance to a specific sample. We introduced the entity SampleCollection as an aggregate of Sample to decouple the mapping from Sample to Data and handle data instances that merge information coming from multiple samples (see Figure 2). It has a role analogous to PatientCollection for Patient.

In selected cases, new data insertion may require additional operations and modifications inside the database. We have developed an abstract Java class loosely based on the Command design pattern to handle such situations. The class contains three methods: *check, retrieve *and *recovery. Check *is executed before inserting the new data, to verify whether all the required conditions (for data submission) are met/satisfied. The *check *method may also be used any time an event - and the related data instance - depends on previous events (as stated by a protocol workflow or pipeline), to verify that all previous required events have been registered in the repository. *Execute *is called immediately after the new data insertion to apply all the additional modifications to database entities. It may also contain a procedure to automatically populate a metadata instance parsing a file header, without need for the user to load them manually using the web form. *Recovery *is run if the *execute *step fails for any reason, in order to fall back to the original configuration. This abstract Command class can be extended to handle different situations. We will show an implementation of it to manage sample aliquot deliveries in the next paragraph.

We newly designed a flexible search interface that allows users to compose queries based on the custom-defined metadata attributes and run them on the database and on the grid, to recover patient and sample information, and files. The model is sufficiently flexible and extensible to encompass various use cases and research studies in Biomedical Science. In the following paragraph we detail a practical application of it in and integrated biobanking scenario.

### Integrated Digital Biobank use case

**Figure 4:**
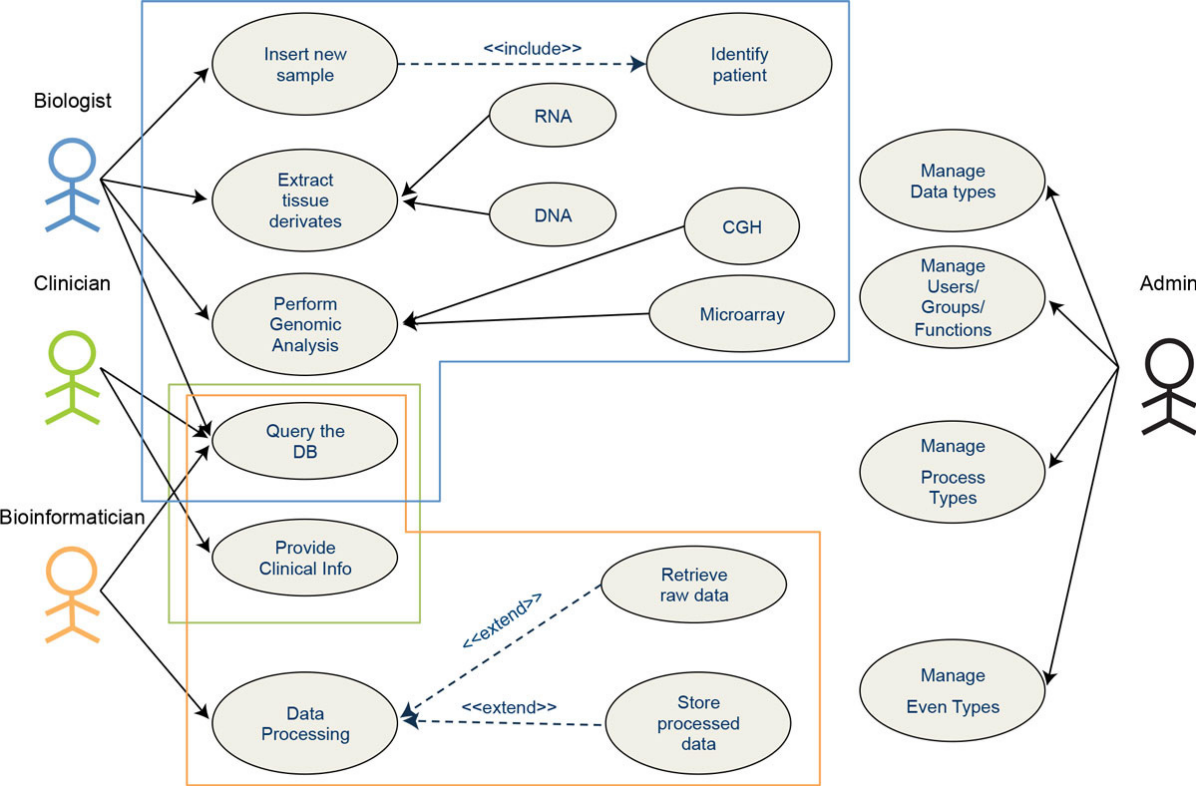
**Use case diagram for the biobanking activity at Gaslini Hospital**. We have identified four actors in the Biobank Management system. The biologist banks tissue and blood samples, extracts RNA and DNA from them and performs structural and expression genomic analyses. The clinician periodically provides clinical data about patients. The bioinformatician retrieves the collected information and processes it using classifiers and machine learning tools. The administrator manages users, groups and functions; creates and activates functions and data types for specific groups. In a small lab or group another actor (e.g. biologist) may also have administrative role.

We have customized and applied our data model to manage the activity of the Biobank Integrating Tissue-omics (BIT) at the laboratory of Molecular Biology of the Institute Giannina Gaslini (IGG). BIT collects tissue and blood samples of paediatric patients and centralizes neuroblastic tumours from all over Italy. The biobank was founded in 2009 and, as of June 2013, more than 1,800 different samples are stored inside the biobank. We have histopathological and genomic characterization of the samples, including structural alterations in DNA (CGH array) and gene expression profiles (Affymetrix DNA Microarray) of about 150 neuroblastoma tumours. The clinical history of the neuroblastoma patients is updated every year. All this information - samples, clinical, genomic and personal data - must be integrated and stored inside the biobank database. Figure 4 details the use-case diagram with all the activity performed inside the biobank.

Each sample must be associated to a patient, univocally identified by name, surname and birth date. For any banked sample, the pathologist provides the histopathological diagnosis and other pertinent information such as the percentage of tumour cells and the biologist extracts nucleic acid derivatives. Multiple extractions from the same tumour can be performed. The tissue and its derivatives are stored and preserved in the biobank for further use.

We have newly designed the sample management system within XTENS to handle multiple derivatives extractions from tissue and blood samples. A patient has one or more samples, and in addition each tissue or blood sample may generate 'children' samples as in the case of genomic derivatives. Use of a foreign key pointing to the 'parent sample' in the Sample entity allows to track each final product or aliquot to the master sample. This way we can also handle multiple steps of sample fragmentation and purification. We have separated personal sensitive information from the remaining patient data into two different entities (i.e. database tables) to guarantee pseudononymization. Only authorized users may access the unique ID that allows retracing and retrieving personal information and link it to clinical data and samples. Each molecular or bioinformatical analysis performed in the biobank is associated to a new data instance registered in the repository.

**Figure 5:**
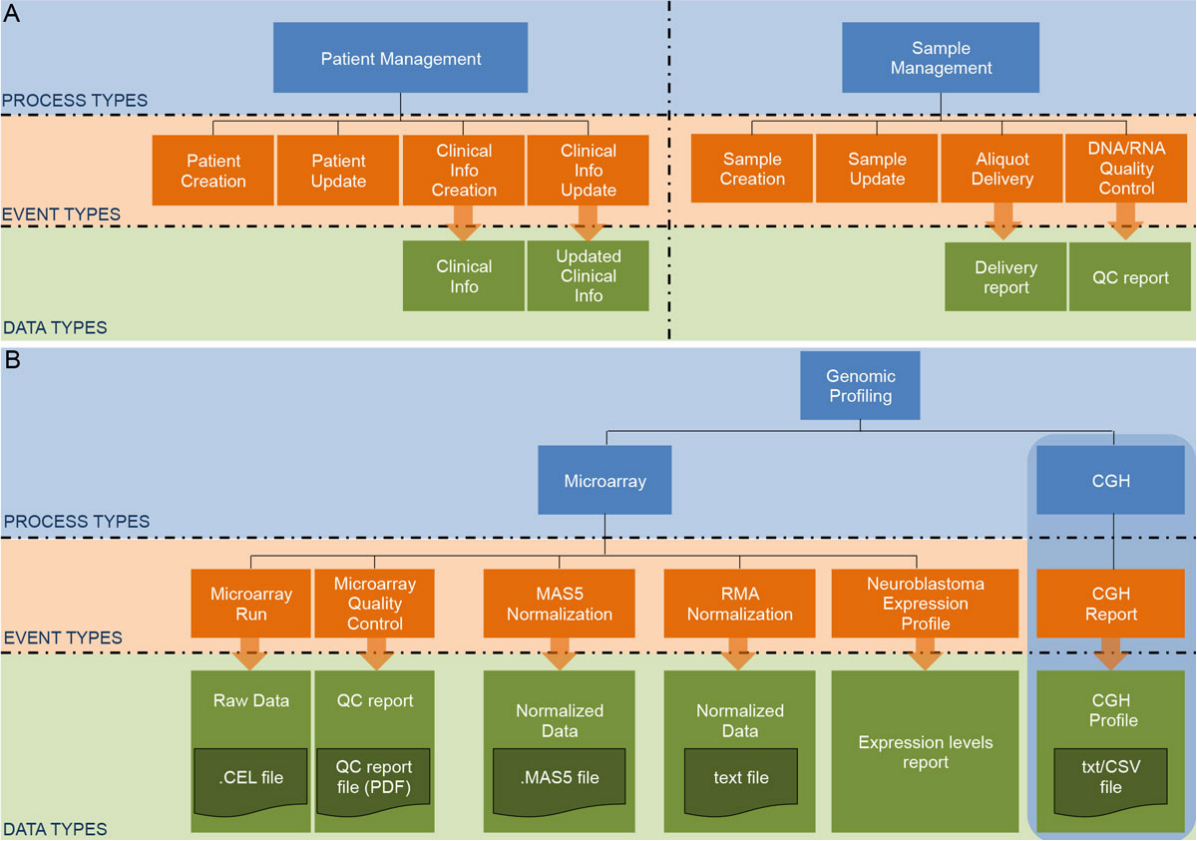
**Detailed process-event schema for Gaslini BIT data management**. The biobank management system consists of the main process types: (A-left) 'Patient Management', (A-right) 'Sample Management', and (B) 'Genomic Analyses'. Through 'Patient Management' we track patient creation and update together and Clinical Information periodical update. 'Sample Management' provides us events for sample creation and update, aliquot delivery and for storing quality control reports on DNA/RNA extractions. Each data instance is described by a set of metadata customized on the basis of SOP requirements and standards. 'Genomic Profiling' is made of two sub-processes to track each step of Microarray and CGH analyses, and further processing steps. Only the Microarray process type is fully detailed in the picture.

According to the process-event schema we have identified three main process types: patient management, sample management and genomic analysis. Through Patient management we track patient creation, modification and deletion and any periodical insertion and update to the Clinical Data provided by Physicians. Sample Management comprises, besides sample insertion and update, also aliquot deliveries of tissue derivatives to other labs or institutions outside IGG for specific genomic analyses or research collaborations. We currently perform two genomic analyses: CGH Array and cDNA Microarray, in our IGG facilities. We created a specific sub-process type for each of them with a full set of event types to track the whole processing pipeline. Details of the Microarray analysis workflow are shown in Figure 5. We store the raw data as .CEL files, and we performed two different microarray normalizations (MAS5 or RMA [39,40]) depending on needs. We also store reports about a set of outcome and prognostic feature predictors, developed at IGG, and based on two machine learning classifiers: (I) a multilayer perceptron neural network [41] and (II) a Logic Learning Machine (LLM) algorithm implemented with the RULEX software [42]. The CGH data pipeline follows an analogous pattern (details are not shown in Figure 5).

We have used the general purpose module inspired by the Command design pattern described in the previous paragraph to update stored material following deliveries of part of it to other labs or institutions. In this scenario, the *check *method controls the selected sample quantity and compares it with the quantity to be delivered. If the latter is greater an error message is returned and the procedure stops. Otherwise, a new sample delivery event and related data are registered in the database and the *execute *method updates the remaining quantity. If any error occurs during the new data registration, the *recovery *method restores the previous quantity value.

**Figure 6:**
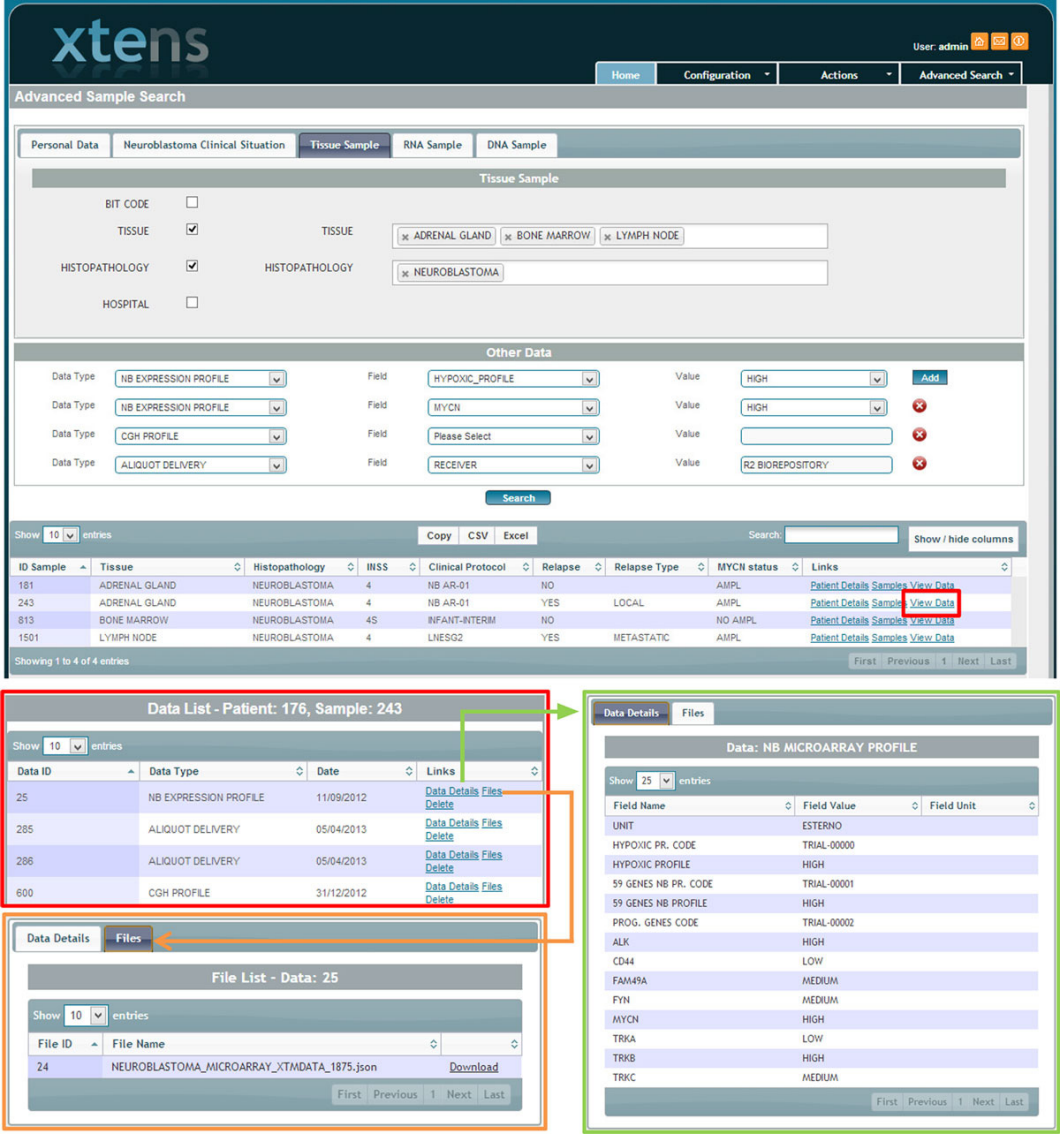
**Digital Biobank sample search form**. The Digital Biobank search form interface provides users with a flexible tool to query their datasets and retrieve stored information and files. It provides a unified view on sample, clinical and genomic information. See Additional File 4 for more information's on the platform capabilities.

We have fully customized the XTENS research page, to handle complex searches within the digital biobanks. Searches can be performed either on patients or tissue/fluid samples, depending on the operator necessities. For sake of simplicity we illustrate the sample search, which is the more detailed of the two, since it contains all patient data as well. The search page is a multi-tab form, where search fields are divided on five main topics: patient info, clinical info (mostly specific to neuroblastoma), tissue sample, DNA and RNA. Each tab contains a full list of fields; using a checkbox list users select the fields they want to be shown in the result table. For each selected item they can specify one or more values for the query. Additional tuning can be performed using the custom-defined data types and metadata fields described as described in the 'Data Model Implementation' paragraph. The result provides an integrated view on all the requested information. For each sample the full list of stored data instances can be visualised, and for any data instance the set of associated metadata and files. Authorized users can export the result as CSV or EXCEL file and download files stored in the data grid. External applications can access data and files using a RESTful web service interface. Details of the search form are shown in Figure 6, where we perform an integrated query based on sample management (i.e. aliquot deliveries) and genomic information (microarray and CGH profiling). Authorized users can create specific data types using the graphical form or submitting a JSON schema via web service. The flexibility of the data type creation allows compliance to various standards. Patient Health Records are tracked as a temporal sequence of events, within one or more processes; each event has a data instance with a user-defined set of metadata to describe it. Thus, the operator can create data types relative to clinical events, such as a tumour onset and relapse, alongside genomic data types, such as MAGE standard format [43]. An example of a MAGE data type creation and data usage is shown in Additional file 1. Users can compose queries based on all the previously defined metadata fields through the graphical query form. Details of an integrated query on patient clinical records and MAGE metadata are shown in Additional file 2.

The Digital Biobank platform has currently been used in production for four months. We have inserted over 1800 primary tissue samples together with all extracted DNA and RNA derivatives form over 1000 patients. We have written a set of Java procedures to retrieve information from spreadsheets and automatically populate database entities, to automatize time-consuming operations such as the initial data import from CSV/EXCEL files and the periodical update of clinical data.

We tested the database performances using a fixed set of queries on tissue samples with multiple table joins. These queries are built with 4 to 6 different data and metadata parameters. Then, we used a MySQL database, populated with about 10,000 data instances, 120,000 metadata, and hosted on a 64- bit computer equipped with 3.5 GiB of RAM. We identified a set of table indexes to optimize the test searches, and we were able to reduce the query time of two orders of magnitude from an average of 178 s down to an average of 0.9 s. As a consequence of indexing, new data insert speed is 31% slower. The system's performance is more than suitable for the current workload. However, it can be improved by more tailored indexing, query caching, and possibly by alternative tuning of MySQL configuration properties, and this strategies will be particularly useful if the dataset scales up. (see Additional file 3 for details).

### iRODS system implementation

All the Digital Biobank Platform is currently hosted on a FUJITSU ESPRIMO P400 personal computer located at IGG, equipped with Ubuntu Linux 12.04. We set up on the same machine an iRODS Zone (i.e. System), named *iggZone*, with a single metadata catalogue (iCAT)-enabled server and two resources: *mainResc*, located in vault within the PC hard disk, and *bakResc*, located in a vault within a USB external hard drive. A single user, currently called *xtens *is enabled for users accessing iRODS from the Digital Biobank portal. All the files uploaded from XTENS are stored inside the sub- collection (i.e. subdirectory) *xtens-repo *and further divided depending on the project and patient identifiers. We have modified an iRODS system rule for file management after submission, called *acPostProcessForPut*, to check file integrity and automatically manage replication of data on the two resources for all the files saved in iRODS using the Digital Biobank portal.

acPostProcForPut {ON($objPath like "/xtensZone/home/xtens/xtens-repo/*")

{msiSysChksumDataObj; msiSysReplDataObj("mainResc","bakResc"); } }

**Figure 7:**
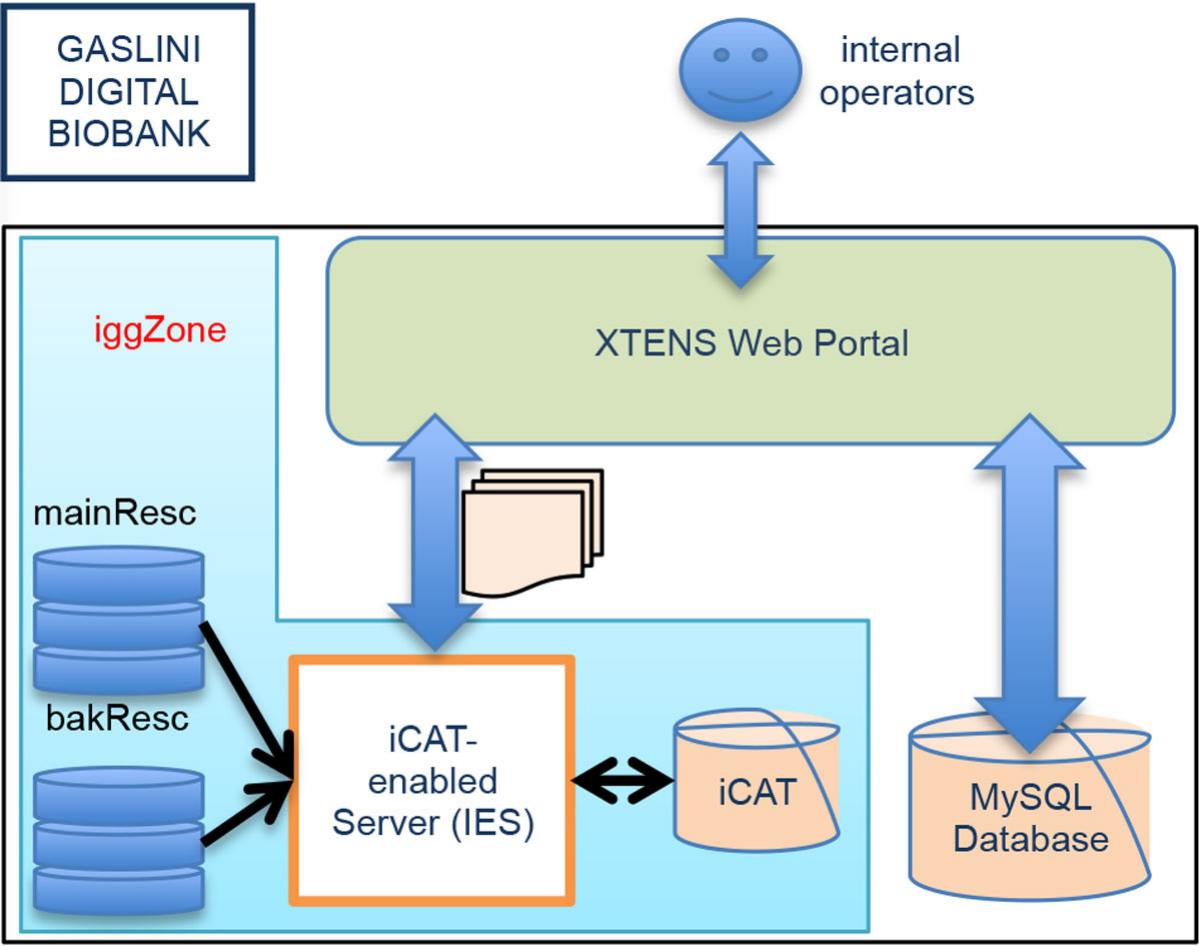
**IGG Digital Biobank Infrastructure**. The three components of the IGG Digital Biobank are deployed on a single computer housed in our IGG facilities. Using the XTENS web portal, the user can insert and query patients, samples and data information on the local MySQL database. They can also store or retrieve files from the data grid, managed by iRODS. The iRODS system - named *iggZone - *consists of an iCAT-enabled server and two distinct resources for safe data replication.

The details of the IGG Digital Biobank Infrastructure are shown in Figure 7.

Since our goal is to use the data grid to share data files and related metadata with other institutions for research purposes we set-up a test bed at DIBRIS to test the XTENS repository with distributed grid storage. To ensure sharing and preservation of data for research activity, we are setting up a prototype of distributed data storage system based on an iRODS Zone, named *dibrisZone*, consisting of three iRODS servers. We have deployed the iCAT-Enabled Server (IES), logically named 'iRODS1', and a second server without iCAT, named 'iRODS2' on two Ubuntu Linux 64-bit machines at DIBRIS. A third server without iCAT, named 'igg-grid03' is deployed on a ProLiant D3 machine at IGG. We are currently working on setting up a secure connection via VPN-IPSec protocol between IGG and DIBRIS using 'iRODS2' and 'igg-grid03' as gateways. So far we have done some tests on the two iRODS servers at DIBRIS using an XTENS test repository deployed on the same machine where iRODS2 resides. iRODS1 has a 'Unix File System' resource named *mainResc01 *for direct access to the user while iRODS2 has a 'Unix File System' resource named *backResc02 *for safe replica of the data. A delayed rule has been set to daily perform a consistency check on the full iRODS file system and repair any broken data.

Once we enable the connection between DIBRIS and IGG we will use the *backResc01 *to replicate all files stored in the 'igg-grid03' server. All the metadata stored inside the biobank MySQL internal database will also be replicated on the iCAT as AVU triples, thus allowing users to perform query based on the custom-defined metadata on iRODS to retrieve files. The DIBRIS-IGG setup will be used as a test-bed for data and file sharing across distinct institutions. Both our iRODS installations - *iggZone *and *dibrisZone *- run iRODS 3.2; *iggZone *is equipped with a PostgreSQL 9.0 iCAT, while *dibrisZone *with MySQL 5.5.

## Discussion

We implemented a platform and data model that offers three main advantages, relative to data management. First, the process-event model can manage both clinical visits/events histories and genomic experiments (with subsequent analyses and post-processing steps) in a uniform yet fluid way. Second, researchers and clinical operators can define new data types and describe them with customized metadata using a graphical web form, without dealing with JSON or XML formats directly, thus not requiring the help of a computer science expert. Third, we provide a user-friendly interface with a data grid system, which can easily scale-up to manage huge files such as high-resolution clinical images or whole genome sequencing data.

We have moved from the previous XML schema to the new one written in JSON, for a number of reasons. First of all, JSON is a lightweight and human readable format, when well structured and indented. Moreover, JavaScript is the natural scripting language on all major web browsers; the JSON metadata schema can be parsed and processed on the client side without loading the server with additional processing and computation. The JSON schema properties, as outlined in Figure 3, can also be easily extended and/or modified according to the community needs, without requiring additional risky and error-prone compilation tasks. If we used XML we would need a tool or library, such as JIBX [44] to bind the XML metadata schema to the Java objects to be manipulated by the code running on the server. This operation would require additional recompilation steps. Finally, we deem that JSON is better suited as a data and metadata exchange format (while XML is more indicated for document exchange) and in general can be mapped more easily to object-oriented systems such as Java or Python. Overall, the JSON metadata schema we propose is a novel approach to document and describe in a highly flexible but consistent format heterogeneous datasets and information in biomedical science, both for clinical and research support. On an end line note, many key-value based NoSQL databases and document containers such as MongoDB and CouchDB use JSON format for document storage. We plan to test a NoSQL metadata catalogue implementation in the next future, to see whether it can improve our query performance compared to a RDBMS.

We chose to use iRODS as our distributed storage manager because its metadata capability fits our requirements of supporting complex user-defined metadata. Moreover, iRODS is relatively easy and quick to install, and enables flexible data management through the Rule Engine. Through it we can manage and track in a seamless and efficient way both relatively small datasets, such as microarray expressions profiles, and, in a soon-to-be future, larger ones, as whole genome sequences. iRODS is already used in production in various genomic centres and biomedical consortia, such as the Wellcome Trust Sanger Institute [45] and the Services@MediGRID project [46].

We want to emphasize that we are not proposing the JSON metadata model as a possible standard, because we think that a single standard format cannot be achieved with the diversity of tools, instrumentation, protocols that are used in the Medical Science as a whole, let alone the variety of disciplines involved. Our data model enables users to continuously define flexible, ad hoc, and loosely structured metadata, for information sharing in specific research projects and purposes. This can improve sensitively interdisciplinary research collaboration, as suggested by a recent social science study [47]. We have shown and tested in a real life situation, that the model allows high flexibility in data description while providing a minimal structure.

The models previously exposed in the background session [5,21,29,31] represent an excellent solution for information sharing, but they may not be perfectly suited to describe multi-disciplinary data integration. For example, none of them offers the possibility to relate genomic information to patients' clinical history, which is critical to practitioners. Our model basically wants to be a solution for clinical needs as well. The idea is to track patients' clinical history as well as their samples' histories as a sequence of processes and events, storing data about their personal clinical studies, in addition to genomic and imaging data available on a distributed data grid environment. We suppose this kind of data model will be appropriate for the future development of personalized medicine. As we wish to accomplish the needs of clinicians and practitioners, our framework must be easily usable by them. A framework like openBIS for example has a lot of utilities, but in order to make it adaptable to individual use case it is necessary to develop extensions to the base system and to configure the base system including the extensions to the operating environment. Developing a new extension for openBIS requires close communication between the researchers and the software developers, while even people who do not have specific informatics skills can customize the XTENS data model.

The designing of the platform took into consideration security aspects when dealing with sensitive data. Inside the database there is a personal data entity that contains all sensitive records about the patient and that can be requested only by authorized operators. Currently, the operators cannot access the Digital Biobank from outside IGG due to security restrictions. We plan to install an anonymized replica of the Biobank database on a DIBRIS server, for data sharing and integration with other European biobanks in the framework of the European Network for Children's Cancer Research (ENCCA) project. The anonymized database will interface with the same iRODS Zone, so a replication of the data grid environment is not required. All the data that we plan in to share with other communities using the data grid is anonymized and can in no way be traced back to the patient identity. Anyway, iRODS supports Grid Security Infrastructure (GSI) besides secure password access and can be integrated with authentication protocols such as Kerberos [45], if more tight security policies must be satisfied.

## Conclusions

Our aim was to develop a digital repository for heterogeneous data for Integrated Biomedical Research, equipped with an interface to a data grid environment for data tracking, preservation and sharing across institutions.

We used the XTENS digital repository and built a general-purpose metadata model in JSON format on top of it. We tested it against an integrated biobanking scenario where heterogeneous data are to be managed. This is, to our knowledge, the first extensible data model integrated with a data grid storage approach proposed for information sharing and data integration in biobanking and multi-disciplinary biomedical research.

In the future we plan to define a set of process and event types for NGS management and integrate it with an NGS analysis pipeline, to automatize retrieval and storage of data on the Digital Biobank. We will test the digital repository as a data sharing platform among different biobanking institutions, molecular biology labs and computer science centres.

## Competing interests

The authors declare that they have no competing interests.

## Authors' contributions

MI developed the model and the software, deployed the Digital Biobank implementation and wrote a major part of the paper, FM tested the software and wrote part of the manuscript, GA contributed to the manuscript, draw the illustrations and implemented some software extensions for automatic metadata acquisition, MF supervised the software development and revised the manuscript, LV supervised the biobanking implementation, provided feedback on the development and contributed to the manuscript.

All authors read and approved the final manuscript.

## Supplementary Material

Additional file 1 Integration between MAGE-TAB standard data and patient health records in the XTENS repository. the document shows that it is possible to create and manage a new data type, exemplified by the MAGE-TAB format, according to accepted standards in bioinformatics. Click here for file

Additional file 2 Integration between MAGE-TAB standard data and patient health records in the XTENS repository. the document shows that it is possible to perform a query between genomic data, for instance the information stored in the MAGE-TAB standard, and the clinical patient health records in the XTENS repository. Click here for file

Additional file 4 Software availability and demo version details. software availability and demo version details. Click here for file

Additional file 3 Database performance test. two plots show respectively search and insert time in the database, under different table indexing conditions. Click here for file

## Declarations

The publication of this article has been funded by the Italian Ministry of Health research funds 'Ricerca Corrente 2012'.

This article has been published as part of *BMC Genomics *Volume 15 Supplement 5, 2014: Italian Society of Bioinformatics (BITS): Annual Meeting 2013: Genomics. The full contents of the supplement are available online at http://www.biomedcentral.com/bmcgenomics/supplements/15/S3
