# Supplementary material for: A digital repository with an extensible data model for biobanking and genomic analysis management
Source: BMC Genomics. 2014 May 6;15(Suppl 3):S3. doi: 10.1186/1471-2164-15-S3-S3 (PMC4083403; doi:10.1186/1471-2164-15-S3-S3)
Supplement: Additional file 1 — Integration between MAGE-TAB standard data and patient health records in the XTENS repository. the document shows that it is possible to create and manage a new data type, exemplified by the MAGE-TAB format, according to accepted standards in bioinformatics. [file 1471-2164-15-S3-S3-S1.pdf]

Additional file 1

Integration between MAGE-TAB standard data and patient health records in the XTENS repository

The following three figures demonstrate that it is possible to create and manage a new data type, exemplified by the MAGE-TAB format , according to accepted standards in bioinformatics. We present screenshots of the graphical user interface, and explanations of the actions required to insert and display data.

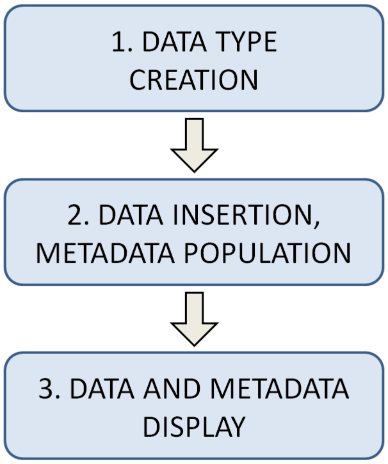

Data management flowchart

1. New data type creation

Figure 1

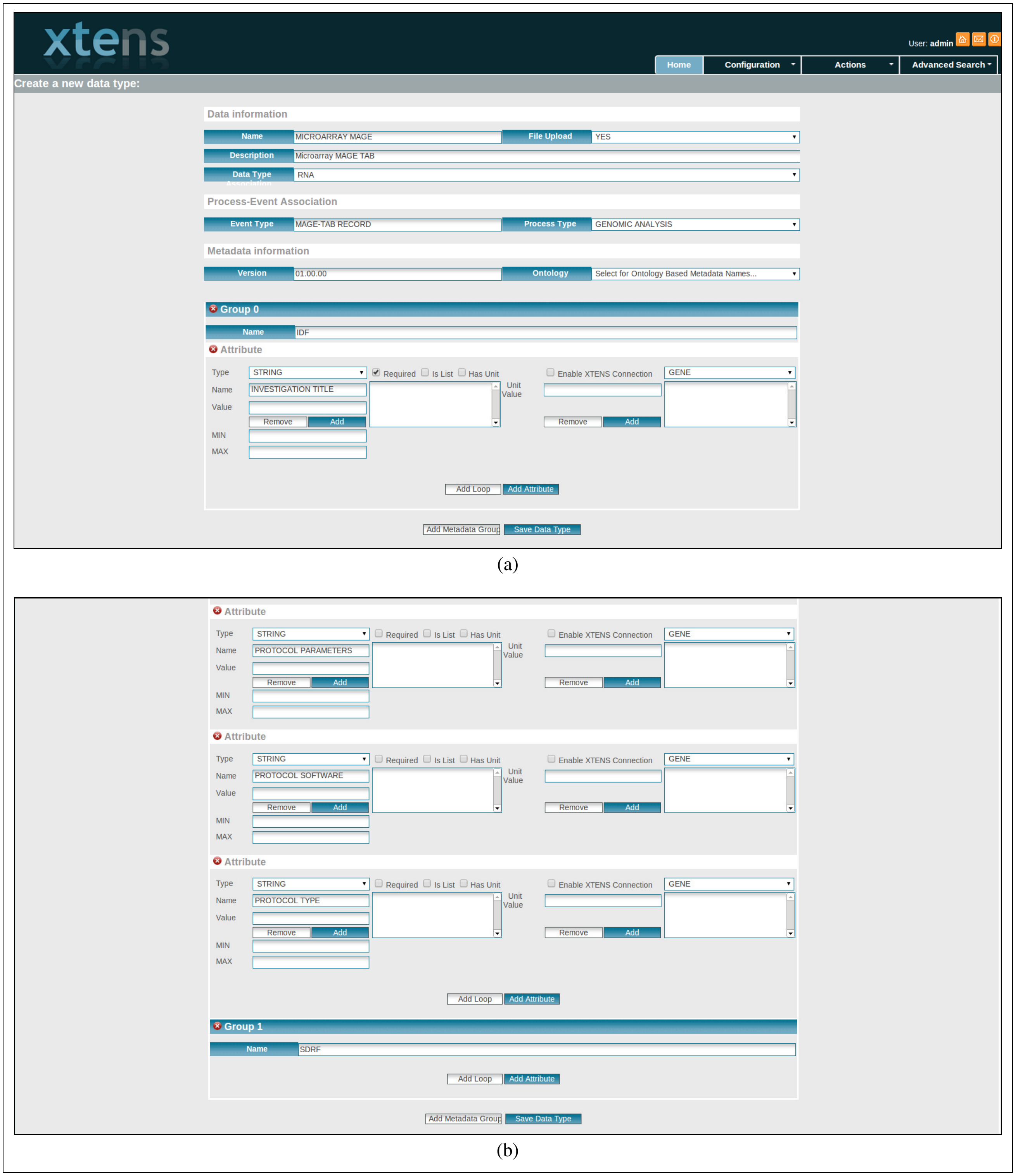

Figure 1 – Creation of a new data type for XTENS repository.

(a) the creation of a new data type for XTENS repository: *MICROARRAY MAGE*, related to the process type *GENOMIC ANALYSIS*. This data type is composed of two metadata groups, according to MAGE-TAB specifics that defines the fields of IDF and SDRF standard files [1] to describe a whole microarray investigation. Following the definition of all the fields in the IDF metadata group (b), SDRF, the second metadata group fields can be created. When all the required metadata groups and fields are defined, the user will click on *Save Data Type* button. A client-side JavaScript procedure will parse the web form and

store all the fields in a JSON metadata schema. The newly created schema is stored in the database and ready to use. Similarly the ADF metadata group could be added (not shown) in case the user would like to describe the design of an array, instead of storing this information in a spreadsheet or in a set of spreadsheets.

2. Insert Data

Figure 2

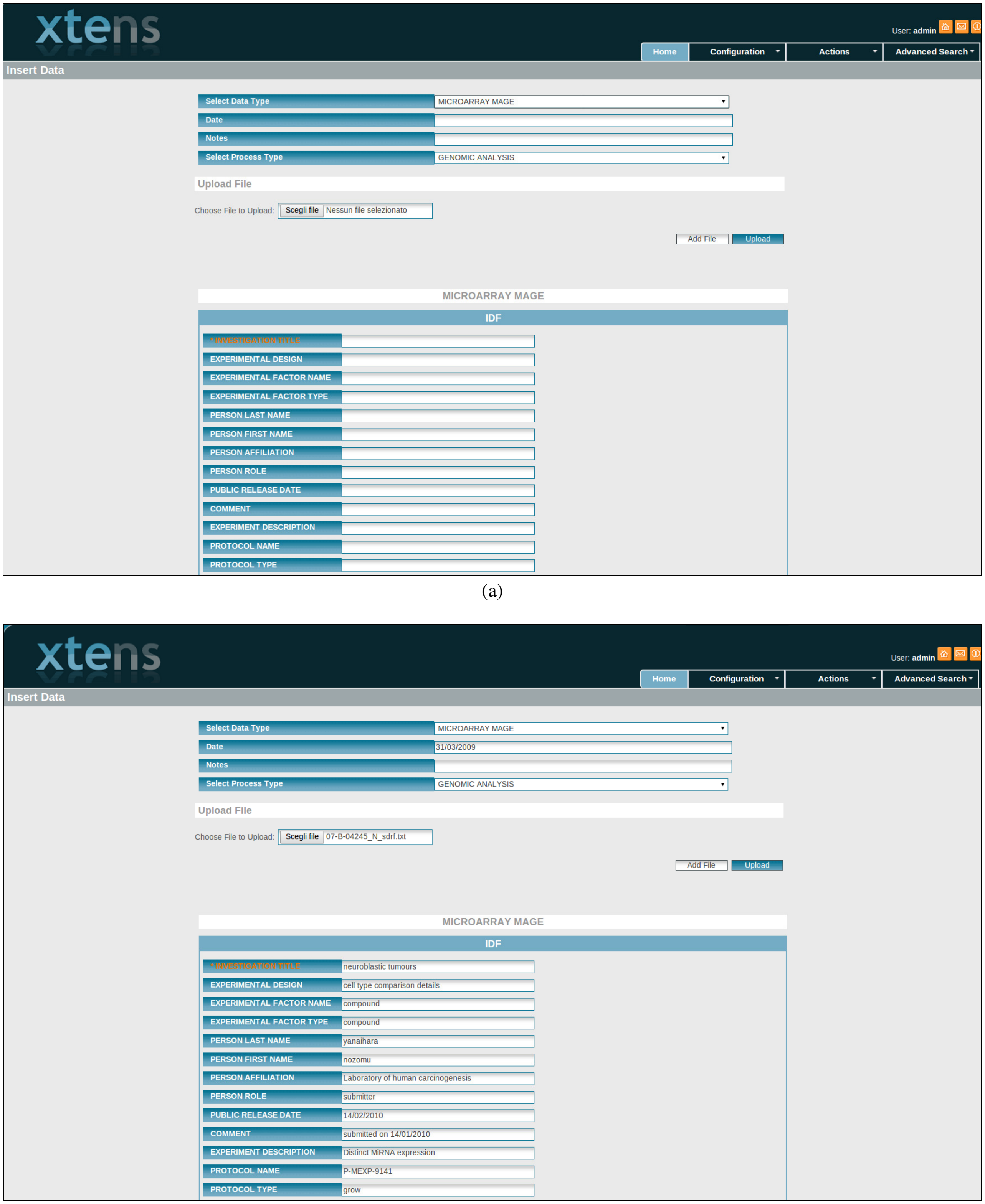

(a)

(b)

Figure 2 – Data insertion for Microarray MAGE data type.

(a) when the option '*MICOROARRAY MAGE'* data type is selected, the application retrieves and parses the corresponding JSON schema, and automatically creates the web form for the insertion of MAGE metadata. The latter are represented by the

fields with the description and the results of a microarray experiment. There is also the possibility to upload a SDRF format file.; (b) a typical insertion is proposed.

3. Display patient record and data details

Figure 3

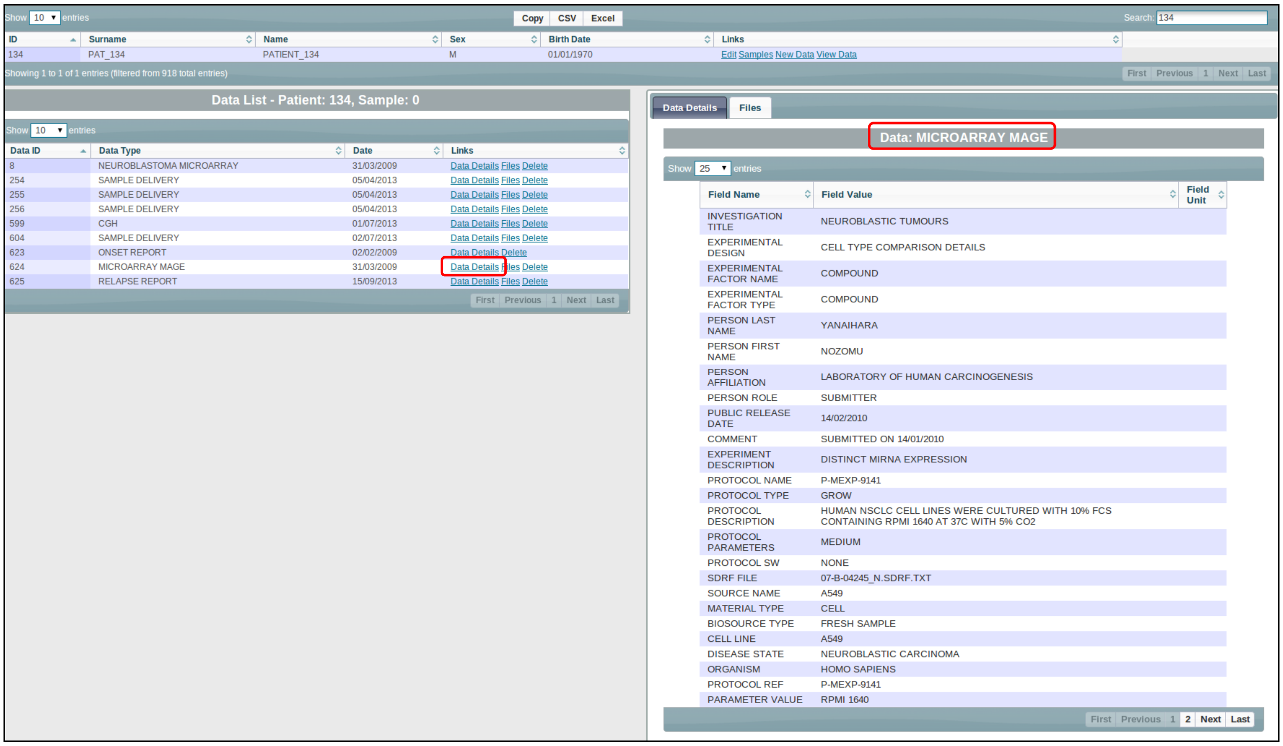

Figure 3 – Example view of microarray MAGE data details.

The screenshot shows a view with all the types of data recorded for a single patient. The list includes clinical data (like *ONSET REPORT* or *RELAPSE REPORT*), data related to sample management (*SAMPLE DELIVERY*) and genomic results (*NEUROBLASTOMA MICROARRAY*, *CGHI*, *MICROARRAY MAGE*). A comprehensive list of the data details is shown as an example in the right panel. If files were uploaded on the Grid, they would be shown in the Files tab.

References

1. Tim F Rayner, Philippe Rocca-Serra, Paul T Spellman, Helen C Causton, Anna Farne, Ele Holloway, Rafael A Irizarry, Junmin Liu, Donald S Maier, Michael Miller, Kjell Petersen, John Quackenbush, Gavin Sherlock, Christian J Stoeckert Jr, Joseph White, Patricia L Whetzel, Farrell Wymore, Helen Parkinson, Ugis Sarkans, Catherine A Ball and Alvis Brazma: **A simple spreadsheet-based, MIAME-supportive format for microarray data: MAGE-TAB.** *BMC Bioinformatics* 2006, 7:489.
