# Supplementary material for: A digital repository with an extensible data model for biobanking and genomic analysis management
Source: BMC Genomics. 2014 May 6;15(Suppl 3):S3. doi: 10.1186/1471-2164-15-S3-S3 (PMC4083403; doi:10.1186/1471-2164-15-S3-S3)
Supplement: Additional file 2 — Integration between MAGE-TAB standard data and patient health records in the XTENS repository. the document shows that it is possible to perform a query between genomic data, for instance the information stored in the MAGE-TAB standard, and the clinical patient health records in the XTENS repository. [file 1471-2164-15-S3-S3-S2.pdf]

Additional file 2

Integration between MAGE-TAB standard data and patient health records in the XTENS repository

The following figures show that it is possible to perform a query between genomic data, for instance the information stored in the MAGE-TAB standard, and the clinical patient health records in the XTENS repository. Screenshots of the graphical user interface, and explanations of the actions required in order to select and display data details, are provided.

Patient advanced search: an example of a query on personal data and MAGE standard data

Figure 1

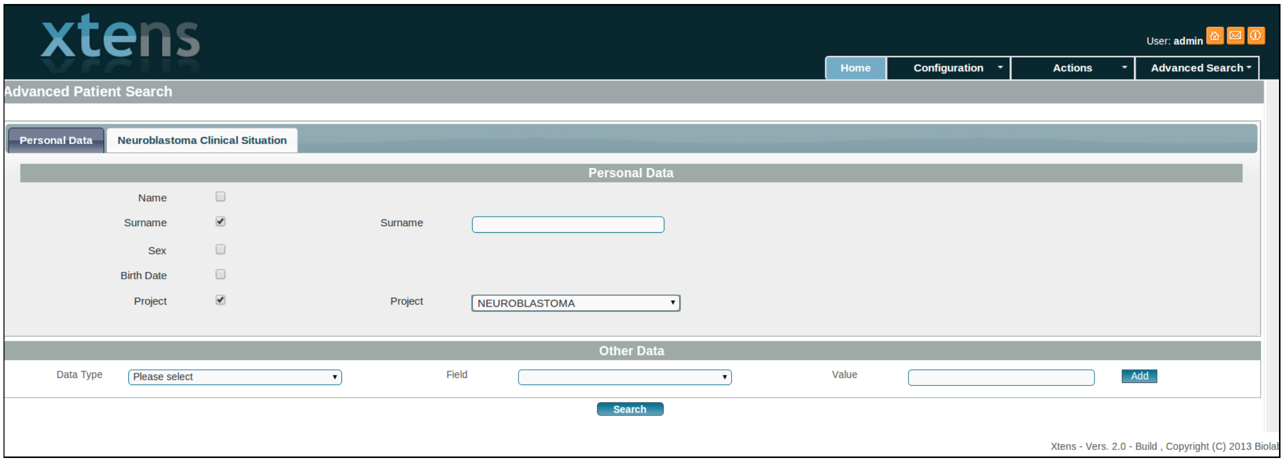

(a)

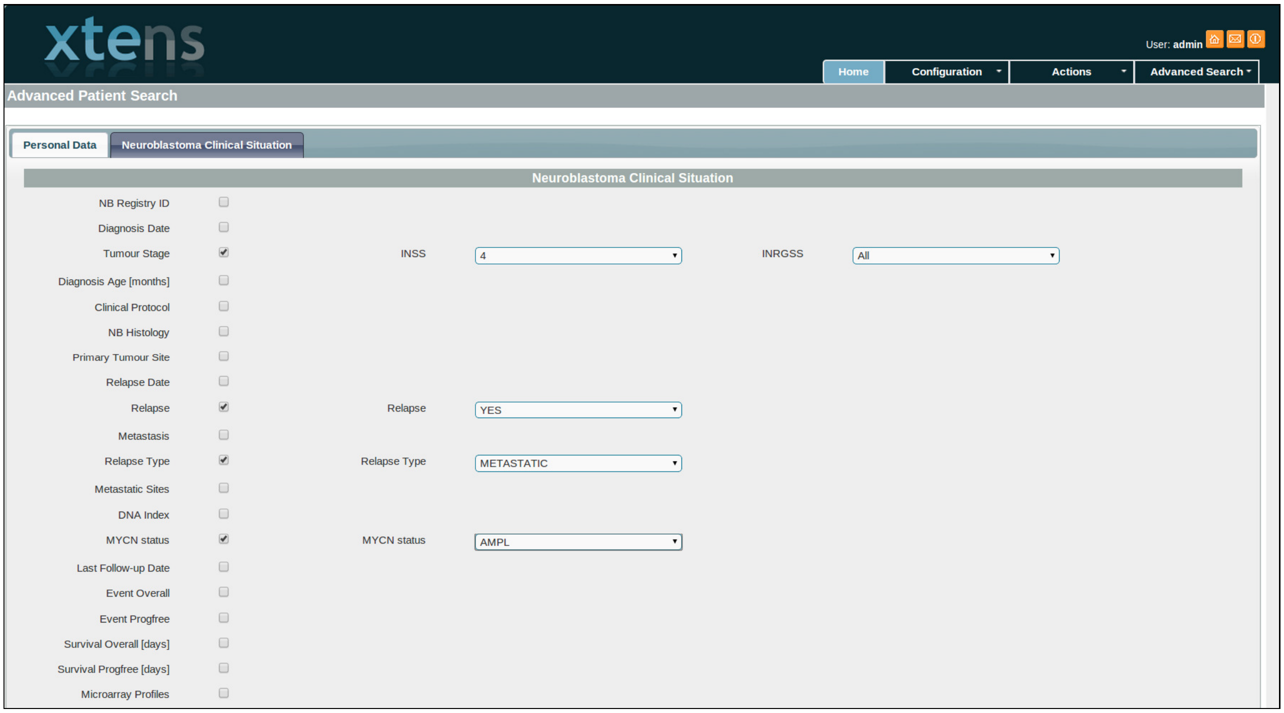

(b)

Figure 1 – the *Advanced Patient Search web form* is composed of two tabs.
(a) *Personal Data* tab: contains options on the personal details of the recorded patients;
(b) *Neuroblastoma Clinical Situation*: contains fields on biological and clinical features characteristic of neuroblastoma tumours.

Figure 2

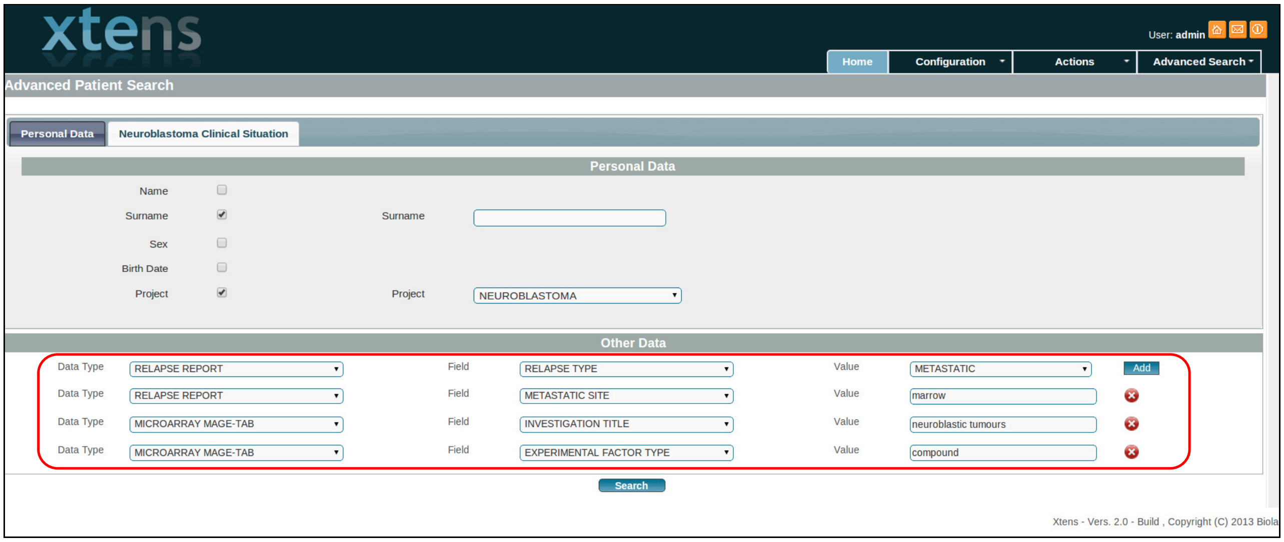

Figure 2 – Selection of specific fields for an advanced query.

The screenshot shows how to select specific fields and values for particular data types. RELAPSE REPORT and MICROARRAY MAGE data types were selected to demostrate that XTENS is suitable to manage patient health records and standard format data.

Figure 3

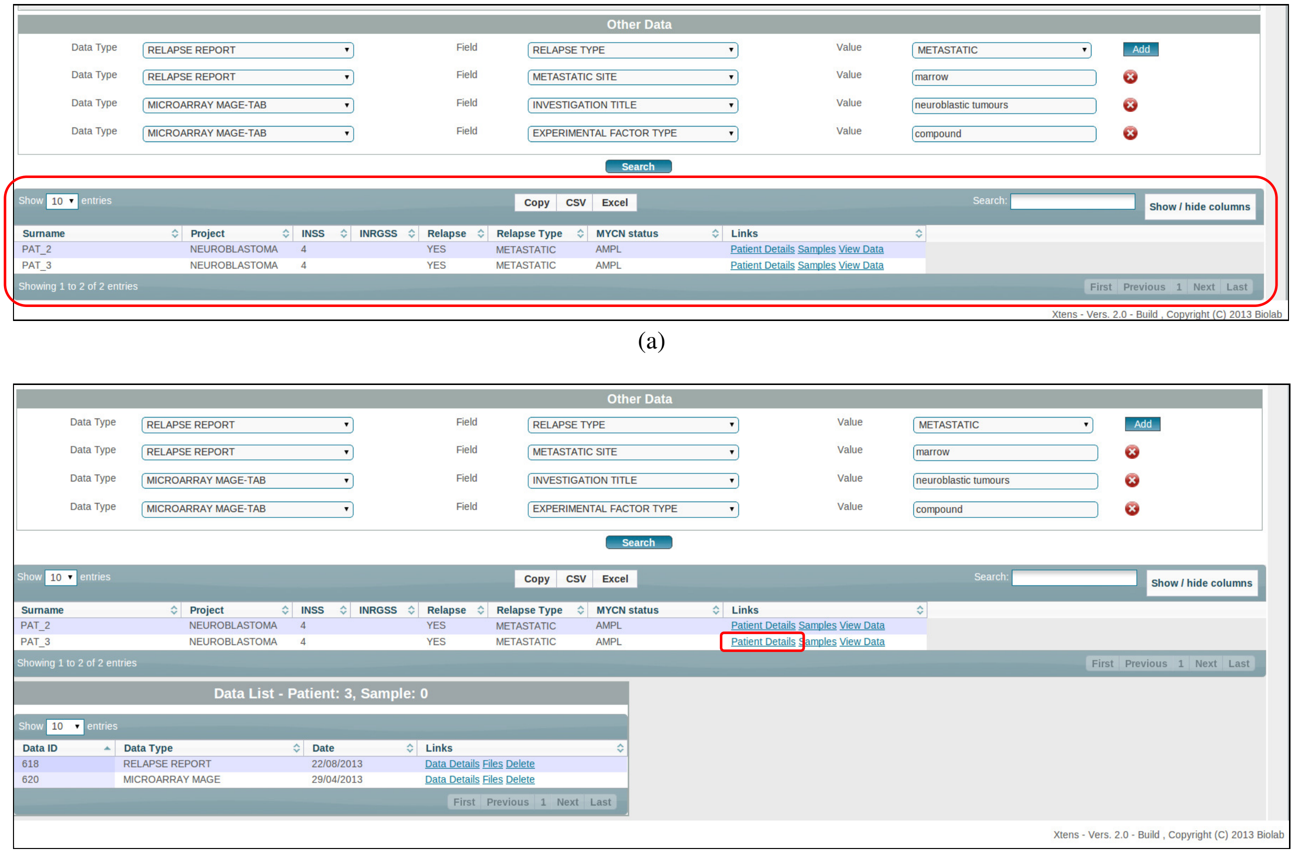

(a)

(b)

Figure 3 – Results of the query of Figure 2.

The screenshot shows the list of patients that satisfy the query criteria.

(a) after the *Search* button is pressed, a list of patients (if any) is displayed; (b) the list of data instances recorded for a single patient (PAT_3 in the example): this list appears after clicking on the *Patient Details* link.

Figure 4

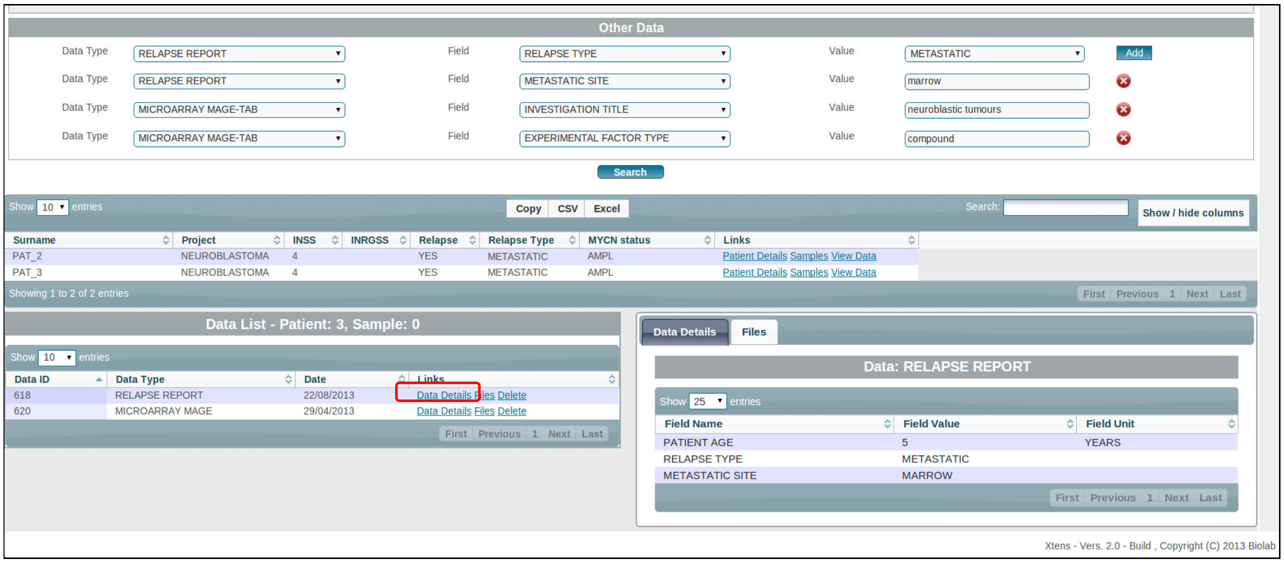

(a)

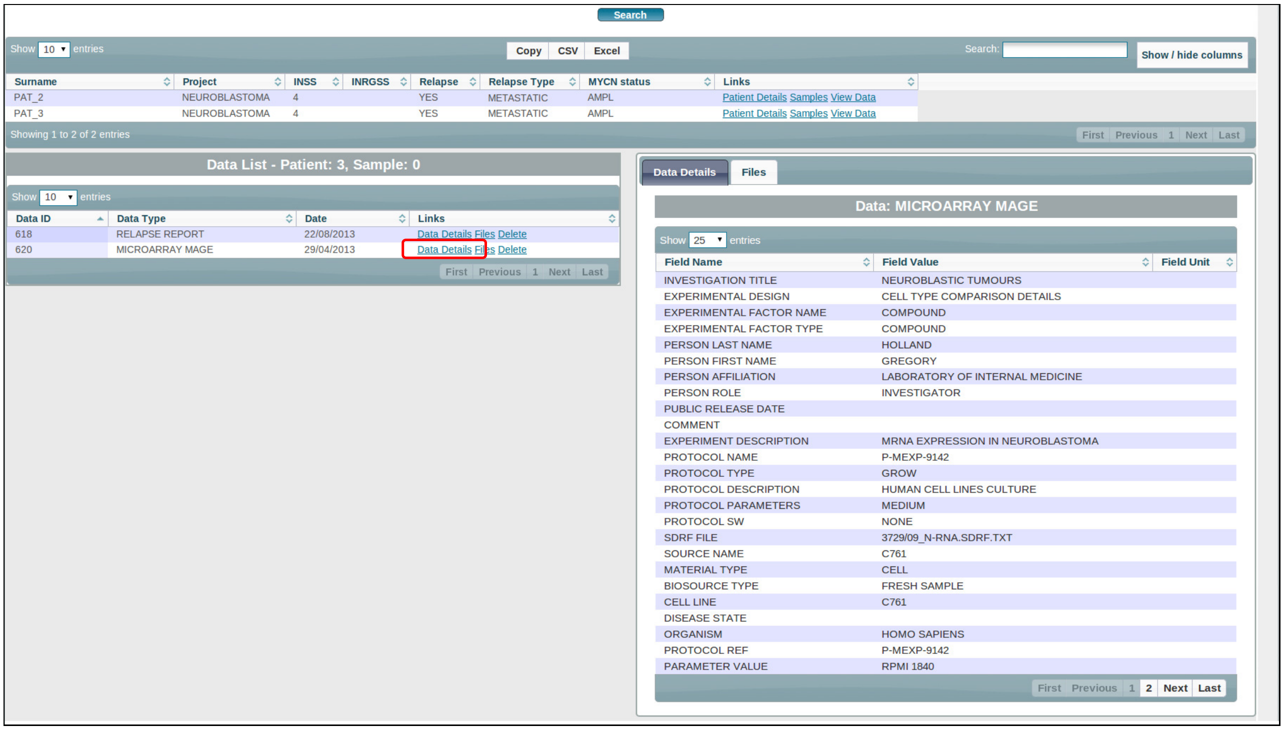

(b)

Figure 4 – Example view of microarray MAGE data details for Patient 3.

The screenshot shows a view with all the types of data recorded for Patient 3. The list includes clinical data (*RELAPSE REPORT*) and genomic results (*MICROARRAY MAGE*). A comprehensive list of the MICROARRAY MAGE data details is shown as an example in the right panel. If files were uploaded on the Grid, they would be shown in the Files tab.
