# Supplementary material for: A digital repository with an extensible data model for biobanking and genomic analysis management
Source: BMC Genomics. 2014 May 6;15(Suppl 3):S3. doi: 10.1186/1471-2164-15-S3-S3 (PMC4083403; doi:10.1186/1471-2164-15-S3-S3)
Supplement: Additional file 4 — Software availability and demo version details. software availability and demo version details. [file 1471-2164-15-S3-S3-S4.pdf]

Additional file 4

Software availability and demo version details

1) Software Availability and requirements

Project name: XTENS

Project home page: http://www.xtens.org

Operating system(s): Unix-like; so far it has been used and tested on Ubuntu Linux

Programming language: Java (server-side code); JavaScript (client-side code)

Other requirements: Java 1.6; iRODS 2.5 or higher

License: To be determined soon. We will release the full code as open source but we are still evaluating all the dependencies to determine the licence type.

Any restrictions to use by non-academics: None; restrictions due to dependencies must be checked yet

2) Demo version online

A demo version of the XTENS-biobank platform is available at http://130.251.10.61:8080/xtens

Users can access with the following credentials:

Username: BMC

Password: BMC2013!!

We have uploaded on Youtube three demos showing the platform and data model functionalities:

1) Biobanking management (Patients, tissues, derivatives…): http://youtu.be/6eA8Um8LIVs

2) Data type creation and management, data registration: http://youtu.be/b9JXaCUnK5Y

3) Advanced sample search example: http://youtu.be/0PndsukylF4

We suggest viewing the demo videos at higher resolution. Users can check the three tutorials we have uploaded on Youtube and try to perform the same actions by themselves or freely explore the platform. For a better experience we recommend users to browse the system using either Google Chrome or Firefox; XTENS has not been fully tested on Internet Explorer yet.
