# Supplementary material for: A digital repository with an extensible data model for biobanking and genomic analysis management
Source: BMC Genomics. 2014 May 6;15(Suppl 3):S3. doi: 10.1186/1471-2164-15-S3-S3 (PMC4083403; doi:10.1186/1471-2164-15-S3-S3)
Supplement: Additional file 3 — Database performance test. two plots show respectively search and insert time in the database, under different table indexing conditions. [file 1471-2164-15-S3-S3-S3.pdf]

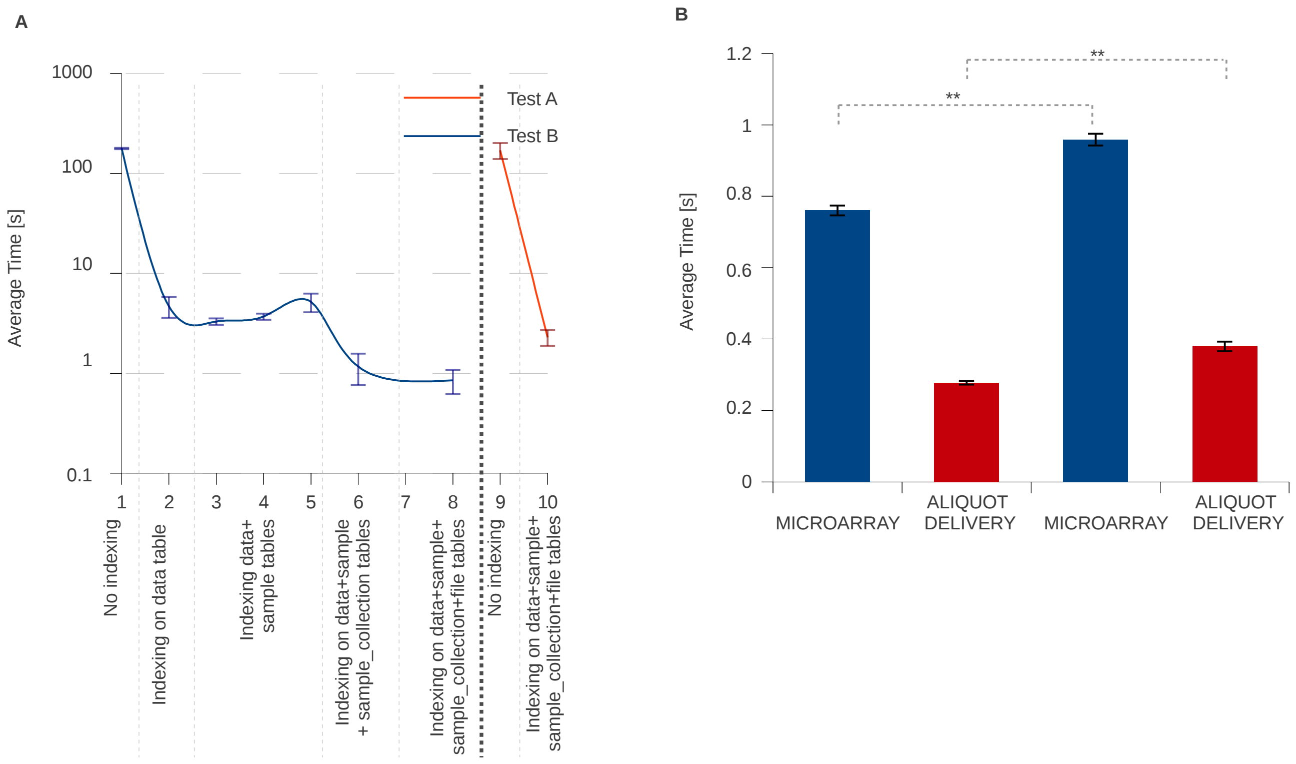

Database performance tests. (A) Search time plotted against different indexing conditions. First no index is used, then we progressively added indexes on tables. TAST A is performed with 1000 patients, 3300 samples, 6200 data instances, and 70.000 metadata. By adding 4 indexes, we were able two reduce the search time from 180s to 0.9s. TEST B is performed on a larger database with the same number of patients and samples and increasing the data and metadata pool to 12.000 and 125.000 records, respectively (B) New data insert average time before and after database indexing. Two different data instances are considered: microarray (in blue, containing 14 metadata) and aliquot delivery (in red, 4 metadata). Insert time is significantly different with p-value < 0.01 (paired t-test). Error bars indicate standard error of the mean.
